# Appendicitis

**Published:** 1910-07-23

**Authors:** 


### SURGERY.

### APPENDICITIS.

### SOME POINTS ABOUT THE NON-OPERATIVE TREATMENT.

So long as inflammation of the appendix remains restricted to that organ but little harm is done to the patient. Indeed, it is doubtful if any of the symptoms of appendicitis, properly so-called, are really due to the inflammation of the vermiform appendix itself; it is much more probable that they only come on when the inflammation has extended to the surrounding peritoneum. This involvement of the peritoneum is the important factor, whether from the point of view of diagnosis or of prognosis. The appendix is only important in so far as it is the original cause of the peritonitis, and therefore its removal may be the best way of treating the peritonitis. It will be well, therefore, to consider the functions of the peritoneum when discussing the treatment of appendicitis. They are threefold: the power of absorption, the power of killing organisms, and the power of adhesion of one surface of peritoneum against another when in any way either surface is irritated.

### Types of Peritonitis.

Considering the third of these first, we may by it classify all our cases of suppurative peritonitis into the localised and the unlocalised, according as to whether the peritoneal folds around the infected area have become adherent to one another and shut it off, thereby forming an abscess, or whether the general peritoneal cavity is still in open communication with this inflamed area. Every case of appendicitis is at some period in this latter stage, where the peritonitis is still local but not yet localised. From this either the peritoneal cavity becomes infected all over, causing a general peritonitis, or the inflammation becomes localised as well as being local, with the formation of an appendicular abscess.

### Functions of the Peritoneum.

So far as the other two functions are concerned, the peritoneum shows interesting variations in its different parts. Absorption occurs chiefly in the upper part of the abdomen around the liver, where the bactericidal power is very slight. In the pelvis the conditions are reversed, destruction of organisms is marked, and absorption is but slight or non-existent.

### Death in Appendicitis.

Death from peritonitis is a combination of collapse due to the inflamed peritoneum and sub-peritoneal tissues taking up large quantities of fluid from the circulation, and of a poisoning of the nervous system by toxins, the products of the infecting organism, which are absorbed from the peritoneal cavity.

It is upon an appreciation of these facts that the treatment of appendicitis must be based. If the case be seen within the first twenty-four hours of the first symptom the infection of the peritoneum is so slight that when the infective agent is removed the peritoneum will itself be able to recover: that is to say, the best thing is to remove the appendix. But when these twenty-four hours are passed the peritoneum is certainly involved, probably to a considerable extent, and it is in the stage mentioned above, of being still local but not yet localised. This is the condition in which operation should be avoided. How, then, should the patient be treated? Treatment should aim at getting the peritonitis to localise, and to localise in a part of the body where absorption is slight and bactericidal properties are marked. Also extreme collapse must be prevented. In other words, peristalsis should be inhibited, the products of inflammation should be got into the pelvis, and fluid should be given.

### Absolute Starvation.

So far as the first is concerned, the best way to inhibit peristalsis is to give nothing by the mouth, and, by *nothing*, *absolutely nothing* is meant, not even water. The passage of fluid along the gut is enough to stimulate peristalsis, and this may tear down some newly-formed adhesion and allow the inflammation to spread a little further, and again a little further, till the whole area is infected. To get the products of inflammation into the pelvis, the effects of gravity should be utilised, and the patient must be sat up in bed. In this way abscesses are more likely to form in the lower part of the abdomen, lives are saved, and the most serious of appendicular abscesses, the sub-hepatic or sub-diaphragmatic, are less likely to occur.

### The Fowler Position.

Sitting a patient up is most easily done in a proper hospital bed with a sloping back and a wedge under the mattress to take the knees; but it can be done efficiently with a little care in any single bed by making a pile of pillows behind the patient and putting another under his knees and tying the corners of the last pillow to the top of the frame of the bed by bandages or tape. It is, however, difficult to manage in a double bed, and therefore it is preferable that every patient with appendicitis should be moved into a single bed with a firm mattress.

As to the administration of fluid, the usual way by the mouth has been cut off in order to inhibit peristalsis; therefore fluid should be given in one of the ways described in the recent article on infusion The Hospital, July 2), and as the administration is to be kept up for three or four days rectal infusion is the best.

### Enemata.

There remains the question of enemata. For the same reason that nothing should be given by the mouth, copious enemata should not be used. At the beginning of treatment, however, it is well to open the bowels with a moderate enema, carefully given, and this is particularly useful as rectal infusion is to be used, and the lower gut must be empty. This treatment, which was first suggested and employed by Oschner, of Chicago, will give the best chances of recovery in all cases of appendicitis that are not operated on, but will be found particularly valuable in those cases of local but not yet localised peritonitis, in which operation by moving the guts and spreading the infection to other parts is so dangerous.

### The Question of Prognosis.

How, then, are these cases to be recognised? If after the first twenty-four hours of the onset of symptoms there is neither general peritonitis nor signs of a localised abscess, the case should be treated as above. In a few days either an abscess will localise and become evident, or the case will quiet down and recover without the need of operation. The difficult cases are those where there is a localised rigidity of the abdominal wall without any prominence; it is then very hard to tell whether this rigidity is due to an abscess or to a local peritonitis. Attention should be paid to the edge where the rigidity passes off to the supple abdomen: if the fingers can dip round the edge into the abdomen it is likely to be an abscess, for in the other case the slightest pressure with the fingers will cause the rigidity to increase in extent and the edge of the rigidity is hard or impossible to determine.

So far no mention has been made of drugs. Opium is of use: it helps to inhibit peristalsis, relieves pain, and so induces sleep. But there is the danger of its masking symptoms, and for that reason, according to many authorities, it should not be given. The difficulty may be got over by giving it hypodermically, in an efficient dose, and by never allowing another dose to be given till the patient has been seen again. Repeated small doses given by the mouth are insufficient to give that complete relief which will allow the patient to sleep, while they are enough to mask any dangerous symptoms which may arise.

